# EXAM: A Framework of Learning Extreme and Moderate Embeddings for Person Re-ID

**DOI:** 10.3390/jimaging7010006

**Published:** 2021-01-07

**Authors:** Guanqiu Qi, Gang Hu, Xiaofei Wang, Neal Mazur, Zhiqin Zhu, Matthew Haner

**Affiliations:** 1 Computer Information Systems Department, State University of New York at Buffalo State, Buffalo, NY 14222, USA; qig@buffalostate.edu (G.Q.); hug@buffalostate.edu (G.H.); mazurnm@buffalostate.edu (N.M.); 2 College of Automation, Chongqing University of Posts and Telecommunications, Chongqing 400065, China; zaiweijian@outlook.com; 3 Department of Mathematics & Computer and Information Science, Mansfield University of Pennsylvania, Mansfield, PA 16933, USA; mhaner@mansfield.edu

**Keywords:** person Re-ID, deep learning, loss function

## Abstract

Person re-identification (Re-ID) is challenging due to host of factors: the variety of human positions, difficulties in aligning bounding boxes, and complex backgrounds, among other factors. This paper proposes a new framework called EXAM (EXtreme And Moderate feature embeddings) for Re-ID tasks. This is done using discriminative feature learning, requiring attention-based guidance during training. Here “Extreme” refers to salient human features and “Moderate” refers to common human features. In this framework, these types of embeddings are calculated by global max-pooling and average-pooling operations respectively; and then, jointly supervised by multiple triplet and cross-entropy loss functions. The processes of deducing attention from learned embeddings and discriminative feature learning are incorporated, and benefit from each other in this end-to-end framework. From the comparative experiments and ablation studies, it is shown that the proposed EXAM is effective, and its learned feature representation reaches state-of-the-art performance.

## 1. Introduction

Person re-identification (Re-ID) has been widely studied to determine whether a person-of-interest has appeared elsewhere, captured by different cameras [1,2,3]. With the widespread use of surveillance systems, finding a match of an image for a particular person in large-scale image and video repositories is difficult because of a myriad of environmental and technical factors, such as variations in illumination, pose, viewpoint, detection and tracking errors, bounding box misalignment, and unpredictable occlusions.

**Figure 1:**
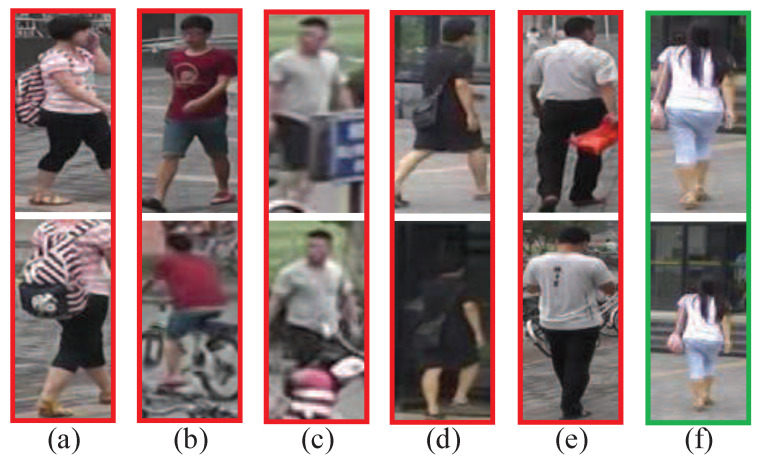
Examples to illustrate the challenges in person RE-ID caused by (**a**) imprecise detection, (**b**) different pedestrian postures, (**c**) occlusion, (**d**) messy background, (**e**) analogous appearance, (**f**) misalignment.

**Figure 2:**
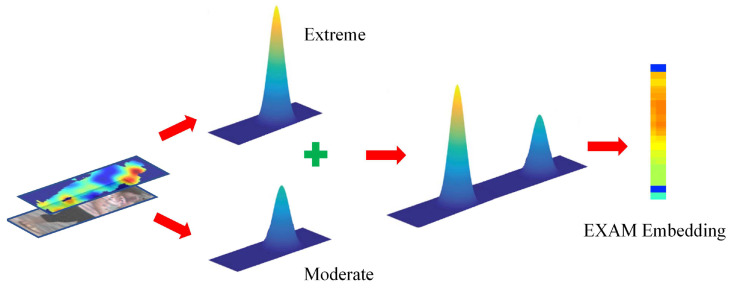
Both Extreme and Moderate features are derived to learn EXAM embeddings.

**Figure 3:**
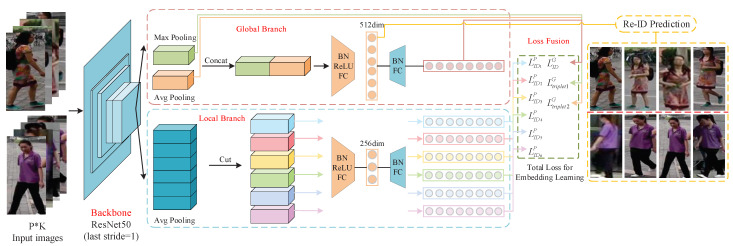
EXAM Network Architecture. It includes four parts: Backbone Network, Global Branch, Local Branch, and Loss fusion. Both global branch and local branch share the same backbone network ResNet-50 to extract the feature maps. The whole network is trained with two triplet losses with batch hard mining and seven cross-entropy losses with label smoothing.

**Figure 4:**
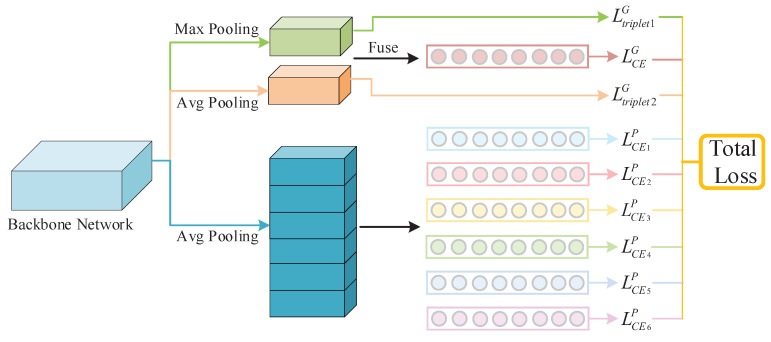
Total Loss Function in the Network.

**Figure 5:**
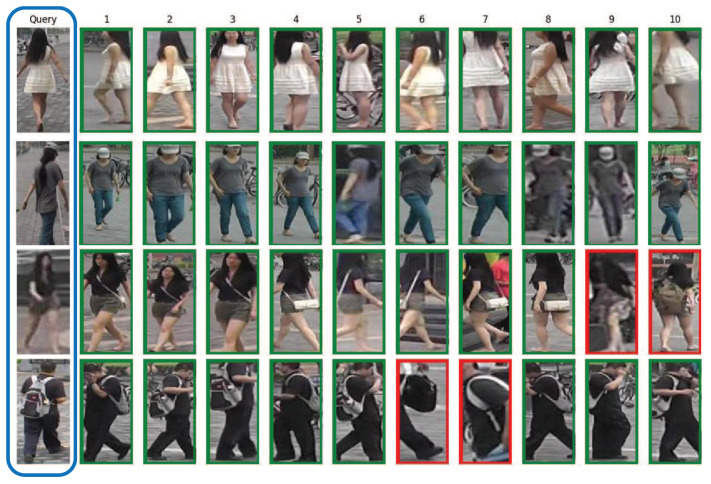
Top-10 ranking list for the query images on the Market-1501 datasets by our proposed method. The pictures with green or red frames indicate the same or different identity as the query image respectively.

The key component of a Re-ID system is feature representation construction. Most early approaches relied on hand-crafted features whose performance is limited due to the gap between the low-level features and high-level semantics [4,5,6]. Recently, deep network-based feature learning has become a common practice in person Re-ID tasks. Deep neural network is originally developed for image classification [7], and its successful global feature learning strategy for classification was directly adopted for the person Re-ID approaches. The learned global representation pays less attention to local details [8], and often suffers weak discriminative ability in identifying targets with similar inter-class common properties or large intra-class differences [9]. For example, the following difficulties are encountered: (1) imprecise pedestrian detection affects global feature learning, e.g., shown in Figure 1a; (2) body posture changes make the learning more difficult, e.g., Figure 1b; (3) unexpected occlusion makes the learned features irrelevant to the human bodies, e.g., Figure 1c; (4) cluttered background or multiple pedestrians with highly similar appearances make the model difficult to distinguish, e.g., Figure 1d,e; (5) Misaligned bounding boxes make the model scale-variant, e.g., Figure 1f.

As a data-driven approach, it is possible for a deep network to learn features from local saliency regions, i.e., guided by some attention-based regularizer during the learning process. At present, one of mainstream Re-ID approaches combines global features with local part-based attention to make the model robust to variations [9,10], in which local features are learned under the visual attentions deduced from the predefined body parts. However, attention derived from partitioned parts alone is not strong enough to supervise the feature learning process. Some alternatives [11] use foreground masks to impose the focus explicitly, but often result in a high risk of having misguided attention at the lower layers due to the poor resolution of input images.

To alleviate this problem, it is better to incorporate the discriminative feature learning and salient attention deducing in an end-to-end network, because they can benefit from each other in the training process [12,13,14]. Thus, in this paper we propose a framework to learn EXtreme And Moderate (EXAM) feature embeddings to deduce the attention at both global and local levels for Re-ID. It may sound oxymoronic to group the two terms “extreme” and “moderate” together. But in fact, they are two inherent aspects of human body appearance: saliency and commonality. Saliency features that are from the most attention attractive visual cues reflect the “extreme” aspects of the body appearance, while “moderate” refers to the common features associated with the concepts of smoothness and consistency without the influence of noise and outliers. If the network can capture both types of attentive information from a person image, the discriminative ability of the learned model would be significantly increased.

The proposed EXAM framework consists of global and local branches sharing a common backbone network based on ResNet-50. Different from conventional global approaches [15,16] learning full body features directly, we apply global max-pooling (GMP) and average-pooling (GAP) operations on feature maps. As shown in Figure 2, conceptually, the extreme and moderate embeddings capture major aspects of body appearance and are integrated to further provide global attentional cues. In the local branch, the entire body is horizontally partitioned into six uniform strips [17], in which the learned local moderate embeddings can provide regional attention cues with suppressed noise caused by target misalignment and background clutter. Finally, in this end-to-end network, a discriminative feature representation is jointly learned under the guidance from both global and local attentions with multiple loss functions. In summary, our contributions are threefold:

1. We propose an extreme and moderate embedding learning framework EXAM for person Re-ID. This is an end-to-end network, providing attention cues to construct discriminative body representations.
2. EXAM has global and local branches. The global extreme and moderate embeddings reflect the saliency and commonality of full human body appearance, while the local moderate embeddings capture the concepts of smoothness and local consistence.
3. By integrating multiple loss functions, the process of deducing attention from EXAM embeddings provides deep supervision for discriminative feature learning. Both procedures are incorporated and benefit from each other.

The rest of this article is organized as follows. Section 2 introduces some related work. The detailed structure of the proposed framework is explained in Section 3. The experimental results are presented and analyzed in Section 4. Finally, the conclusion is drawn in Section 5.

## 2. Related Work

### 2.1. Feature Representation Learning

Conventional methods [4,5,6] use hand-crafted features in person re-ID task, such as color histogram, HOG (Histogram of oriented gradient) and SIFT (Scale invariant feature transform) [4,5,6]. Their performance is limited due to the gap between the low-level features and high-level semantics. Recently, deep learning-based methods have become mainstream in the field of Re-ID. The first deep network approaches for Re-ID were introduced in 2014 [15,16]. Since deep neural networks are originally developed for image classification, its global feature learning strategy for classification was directly adopted in the earlier person Re-ID approaches. For example, Tao et al. [18] proposed a deep multi-view feature learning (DMVFL) scheme to collaborate both hand-crafted and deep features in a simple manner. Zheng et al. [19] proposed an ID-discriminative Embedding (IDE) model, which views the training process of person Re-ID as a multi-class classification problem where each identity is a distinct class. IDE models have been widely adopted in Re-ID community. Compared with hand-crafted methods, deep learning approaches achieved a great improvement in recognition accuracy. However, these learned global representations mainly focuses on full body semantic and pays less attention to local details [8]. It naturally lacks flexible granularity for feature description and often suffers weak discriminative ability in identifying targets with similar inter-class common properties or large intra-class differences [9].

Besides global features, more methods also used human body part information to extract the local feature descriptor for Re-ID performance improvement [20]. There are several ways of obtaining body part information. One is to perform body part estimation by human parsing techniques to find meaningful body parts, such as head, torso, limbs etc., in which well-aligned part features can be extracted. This method usually requires an additional pose detector which may be prone to detection errors due to the gap between the person Re-ID and human pose estimation datasets [10,21]. Alternatively, in [22], a pedestrian image is divided into three regions according to four estimated body key points, and then the local features can be learned from individual regions. Furthermore, some methods directly divide the image into several horizontal partitions as the parts without relying on error-prone estimation algorithms. Part-based Convolutional Baseline (PCB) [17] is a typical approach in this category. It horizontally partitions a person bounding box into several uniform stripes, each of which represents a certain body part. The local features are learned from individual strips and input into its corresponding classifier. The performance of a PCB approach is further improved with a refined part pooling (RPP) strategy to enhance within-part consistency. The experimental results show that the PCB + RPP is effective. How the system integrates multiple parts is essential for organizing local features. Aggregating multiple part-level local features by multiple loss functions [23,24] can guide the network to learn a robust representation for unseen persons.

According to the experimental results, local feature descriptors usually perform better, but valuable global feature information is completely ignored. At present, one of mainstream Re-ID approaches combines global features with local part-based attention to make the model robust to variations [9,10], in which local features are learned under the visual attentions deduced from the predefined body parts.

### 2.2. Attention Cues

Attention information is beneficial for discriminative Re-ID model learning. Its extraction schemes have been widely studied to enhance body appearance representation learning. Usually, attention can be derived from spatial space and different convolutional channels. Within a person image, Harmonious Attention CNN (HA-CNN) model [25] jointly learns the local pixel attention and global regional attention to enhance the robustness of feature representation against misalignment. In [26], a channel-wise Fully Attentional Block (FAB) is designed to adjust the feature response to improve the model discriminability. By introducing both spatial- and channel-wise attention, SCAL [27], a self-critical reinforcement learning framework, achieved state-of-the-art performance on benchmark datasets.

Attention cues can be deduced from local parts feature learning as well. Unlike other spatial and channel-based attention schemas, Chen et al. [28] deploy a high-order polynomial predictor to produce scale maps that contain the high-order statistics (attentions) of convolutional activations. In this way it can capture subtle discriminative features. Similarly, second-order non-local attention is introduced in SONA [12] to directly model long-range relationships. An Interaction-and-Aggregation (IA) [29] models the inter-dependencies between spatial features and aggregates the correlated body part features. However, attention derived from partitioned parts alone is not strong enough to supervise the feature learning process. To eliminate the impact of background clutter, a Mask-Guided Contrastive Attention Model (MGCAM) [11] is designed to use foreground masks to impose the focus explicitly. MGCAM is trained with a region-level triplet loss. However, this approach often results in a high risk of having misguided attention at the lower layers due to the poor resolution of input images. Zhou et al. [30] designed a consistent attention regularizer (CAR) in a feedforward attention network to learn discriminative features from the foreground regions. As a result, the network will focus on the foreground regions at the lower layers, and the network can effectively deal with the target misalignment and background clutter at the higher layers.

From the literature, attention is derived from discriminative [14], diverse [13], low-level [30] and high-order [28] properties of the feature maps. But at least two important inherit aspects of body appearance are missing: saliency and commonality, which are visually attractive to human vision [31]. In this work, we utilize the extreme (saliency) and moderate (commonality) embeddings for attention deducing.

## 3. The Proposed Method

### 3.1. Network Architecture

We propose a Re-ID framework EXAM that learns extreme and moderate embeddings to deduce attention cues for discriminative human appearance feature learning. The overall network structure is depicted in Figure 3. It consists of four major components: a backbone network for low-level feature extraction, a global branch for learning saliency and commonality embeddings from full body appearance, a local branch for learning part-based attention embeddings, and finally, a joint multi-loss deep supervision for simultaneously discovering attention cues and optimizing discriminative feature representation.

Backbone Network: The backbone network learns and extracts the feature maps of pedestrian images. ResNet-50 has demonstrated competitive performance in many vision systems, and has been widely used as the backbone for Re-ID [9,32]. We also adopt ResNet-50 with the pretrained parameters on ImageNet [7] in our approach, with some modifications. Specifically, we remove the last fully connected layer, and add a dimension reduction module and a classification layer for multi-loss training. Since a large spatial view can provide rich feature details, we remove the last down-sampling layer in res_conv5_1 block and change the stride of the last convolutional layer from 2 to 1 to get larger size feature maps. For example, given the input image size 256×128 and the stride value 2, the size of the output feature map is 8×4. If the stride is changed to 1, we can get a feature map with size 16×8. In all of the following experiments, the size of the input image is 288×144. With stride=1, the spatial size of the output feature map is 18×9. This modification improves the model performance, while only adding a small amount of computation cost without introducing an extra burden for parameter training.

Extreme and Moderate Features: Extreme and Moderate embeddings are derived from global max-pooling (GMP) and average-pooling (GAP) respectively. Global Max-pooling performs the feature selection from the 2D feature map, and captures the strongest signal (body saliency) while making the embedding translate-invariant [33]. Average-pooling considers all signals from the feature map, and calculates the mean value, in which noise and outliers can be suppressed, which makes the embeddings robust to pose variation and cluttered backgrounds. Equations (1) and (2) are their formula respectively, where $f_{ch}$ is the feature map of a certain channel, *i* and *j* are the indexes of width *w* and height *h* on the feature map.

(1) $$GMP_{ch}=\max_{0\leq i<w,0\leq j<h}f_{ch}\left(x_{i},y_{j}\right)$$

(2) $$GAP_{ch}=\frac{\sum_{i=0}^{w-1}\sum_{j=0}^{h-1}f_{ch}\left(x_{i},y_{j}\right)}{w\times h}$$

Global Branch: The global branch is connected after the backbone network to learn the extreme and moderate embeddings from full body images. It takes the feature map with the size [1, 2048, 18, 9] from the backbone network. The first dimension 1 represents the number of images; the second value 2048 is total number of channels of the feature map from ResNet-50; the third and fourth values are the spatial height and width of the feature map, representing 18×9. The global branch generates two feature embeddings (vectors) against the full body feature map. The global average pooling (GAP) and global max pooling (GMP) operations are performed on [1, 2048, 18, 9] feature map, to produce two [1, 2048, 1, 1] vectors respectively.During testing phases, both GAP and GMP embeddings are concatenated into a 4096-dimensional vector as the feature representation. This long vector would be followed by a feature reduction module containing a batch normalization layer, a LeakyReLU layer, a fully connected layer to reduce the dimension to 512, and a second set of batch normalization and fully connected layers as the third compact embeddings. Extreme (GMP), moderate (GAP) and the mixture embedding vectors provide meaningful visual attention for discriminative feature learning.

Local Branch: Similar to the PCB approach [17], the entire feature map with the size of [1, 2048, 18, 9] from the backbone network is horizontally partitioned into six uniform strips. The size of each is [1, 2048, 3, 9]. Different from the global branch using two pooling operations on the feature map, only the average-pooling (GAP) operation is applied on individual partitions to get 6 part-based embedding vectors [1, 2048, 1, 1]. After being processed by the dimension reduction module, the final six local part-based 256-dimension embeddings are produced. The local branch extracts moderate embeddings with suppressed noisy information or outliers and deduces the attention cues that bring smoothness and consistence semantics into the feature training process.

### 3.2. Multiple Loss Supervision

In EXAM, multiple cross-entropy loss and triplet loss are combined for embedding and feature representation training, which are mutually beneficial for Re-ID tasks.

Cross-Entropy Loss with Label Smoothing: Cross-entropy loss is commonly used in multi-classification tasks. It is usually placed in the last layer of the classification network to measure the dissatisfaction with the prediction from the current model given the training data. Here, the loss value is calculated by the softmax-based cross-entropy function:

(3) $$L_{soft\max}=-\sum_{i=1}^{N}\log\frac{e^{W_{y_{i}}^{T}f_{i}}}{\sum_{c=1}^{M}e^{W_{c}^{T}f_{i}}}$$

where, *N* and *M* respectively represent the total number of samples and the number of classes in the dataset; $W_{c}$ represents the weight vector for class *c*; and $f_{i}$ refers to an input feature map. Since the data samples of existing Re-ID datasets are not enough, directly using the cross-entropy loss can easily lead the model to over-fitting. So, Label smoothing Regulation (LSR) [34] is used to ease the problem. Thus, the cross-entropy loss with label smoothing is shown in Formula (4):

(4) $$L_{CE}=\left\{\begin{array}{cc} \left(1-\frac{N-1}{N}\varepsilon \right)\times L_{soft\max} & ,if(i=y)\\ \varepsilon /N\times L_{soft\max} & ,if(i\neq y) \end{array}\right.$$

Where ε is a small constant hyperparameter, combined with the dataset size *N* to adjust the loss value during training. When the dataset is small, cross-entropy loss with LSR can significantly inhibit the over-fitting phenomenon of the model.

Triplet Loss with Batch Hard Mining: Essentially, Re-ID can be treated as a retrieval ranking problem, since its goal is to find a target in a dataset which is the best match against a query sample. A triplet loss function can be used for ranking metric learning. The basic idea is that the distance between a positive pair should be smaller than a negative pair by a pre-defined margin. Specifically, the network uses three pictures $\langle D_{i}^{a},D_{i}^{p},D_{i}^{n}\rangle$ as the input to the triple loss, where $D_{i}^{a}$ is the anchor sample, $D_{i}^{p}$ and $D_{i}^{n}$ are the positive (with the same label as the anchor) and the negative samples (with the different label). Then the triplet loss is expressed as:

(5) $$L_{triplet}=\frac{1}{N}\sum_{y_{a}=y_{p}\neq y_{n}}^{a,p,n}{\left[t+d_{a,p}-d_{a,n}\right]}_{+}$$

where *t* indicates a margin between the positive and negative pairs. *N* represents the total number of triples in the whole network, and *d* is the metric distance between two samples.

The regular triplet loss randomly selects a group of triplets from the training data. Usually a random selection consists of easy triplets which would result in the model with weak discriminative ability. To alleviate this issue, batch hard mining [35] is applied to select sample pairs that are hard for the model to discriminate. Specifically, it randomly picks *P* identities and *K* samples from each identity to form a mini-batch set with the size P×K. For each anchor sample $D_{i}^{a}$ in a batch, a positive sample $D_{i}^{p}$ with the largest distance from $D_{i}^{a}$, and a negative sample $D_{i}^{n}$ with the smallest distance from $D_{i}^{a}$ are selected. Then the formula of triplet loss with batch hard mining is as follows:

(6) $$L_{hardtriplet}=\frac{1}{P\times K}\sum_{i=1}^{P\times K}{\left[t+\max D_{a,p}^{+}-\min D_{a,n}^{-}\right]}_{+}$$

Compared with the traditional triplet loss, the triplet loss with batch hard mining focuses on more indistinguishable samples in the dataset during training, and can bring better performance for the Re-ID task.

Total Loss Function of Network: In this framework, multiple loss functions are integrated to complete the network training (Figure 4).

The global extreme and moderate embeddings carry the global attention cues about saliency and generality from the full body respectively. We employ two triplet losses $L_{triplet1}^{G}$, $L_{triplet2}^{G}$ with batch hard mining for both. Additionally, the long vector (GMP+GAP) from the global branch and six moderate embeddings of body partitions are trained by seven Softmax-based cross-entropy loss functions: $L_{CE}^{G}$, $L_{CE_{1}}^{P}$∼$L_{CE_{6}}^{P}$ respectively. Thus, we have a total of nine losses, and perform a weighted linear sum to fuse them as the total loss value:

(7) $$L_{total}=\sum_{i=1}^{9}w_{i}L_{i}$$

where $L_{i}$ refers to one of the nine losses, either the triple or cross-entropy value, and $w_{i}$ is its corresponding weight for fusion. In this work, we used a fixed weighting strategy, empirically set w=0.5 for each triplet function, and w=0.143 for each cross-entropy loss function.

This aggregated loss plays the role of deep supervision to deduce better attention cues, which are incorporated to support the discriminative feature representation learning.

## 4. Experiments

### 4.1. Platform Settings

Implementation details: We resized the input image to 288×144, and used the pre-trained parameters on ImageNet [7] to initialize the backbone network. For data augmentation, training images were horizontally flipped and erased randomly (REA) [36]. For the triplet loss in Equation (6), we set the margin t=0.3, identity size P=8, and samples per identity K=4 respectively for batch hard mining. Therefore, the size of a mini-batch is P×K=32. For the cross-entropy loss with label smoothing in Equation (4), the ε value was set to 0.1. We chose SGD as the optimizer, and set the momentum to 0.9, and the weight decay factor for L2 regularization to 0.0005. In order to improve the learning effectiveness, a warm-up strategy was adopted to start over the network. The total training process has 250 epochs. We set the initial learning rate to 3 × 10${}^{-4}$ and set it to 3 × 10${}^{-2}$ in the first 10 epochs. After 60, 130 and 220 epochs of training, the learning rate was reduced to 3 × 10${}^{-3}$, 3 × 10${}^{-4}$ and 3 × 10${}^{-5}$ respectively. All the experiments in this work followed the same settings described above. We trained and tested the model on a PC (Intel^®^Xeon^®^CPU E5-2667, 256 GB RAM) with one Nvidia Tesla P100 16 GB GPU. It took about 24 h to train the EXAM model.

Evaluation metrics: To compare the Re-ID performance with other methods, we evaluated all approaches following standard protocols on benchmark datasets, and used the Cumulative Matching Characteristics (CMC) at Rank-1, Rank-5 and Rank-10 and mean Average Precision (mAP) on the testing datasets. All the results were obtained in a single-query setting, and the re-ranking optimization algorithm was not used.

### 4.2. Datasets

Three publicly available benchmark datasets were used for evaluation.

Market-1501: This dataset includes 32,668 outdoor images of 1501 persons. During dataset collection, a total of six cameras were placed in front of a supermarket. There are 751 identities with 12,936 images in the training set; and 750 identities with 3368 query images and 19,732 gallery images in the testing set. The pedestrian detection bounding-boxes of query images are drawn manually, while the bounding-boxes of the gallery images are detected by a DPM detector [37].

DukeMTMC-reID: This dataset has 36,411 outdoor images of 1404 persons taken by 8 synchronized cameras on the Duke University campus. The training set has 16,522 images from 702 identities, and the testing set has 19,889 images from other 702 identities. Within the testing set, there are 2228 query images and 17,661 gallery images. The detection bounding boxes were semi-automatically generated, i.e. detected by DPM first, and then, adjusted manually.

CUHK03: This dataset contains 14,097 outdoor images of 1467 identities shot by six surveillance cameras at the Chinese University of Hong Kong(CUHK) campus, where 767 identities with 7368 images are in the training set. There are two ways to annotate a bounding-box for this dataset, manually labeled pedestrian bounding boxes and automatic detections by a DPM detector. We conducted experiments on both types of bounding-boxes.

All images from these datasets are from outdoor scenarios. As compared with indoor scenarios, the person Re-ID task is usually more challenging in the outdoor environment because of more diverse pedestrians, a chaotic environment and unstable lighting conditions caused by weather changes, sun directions, and shadow distributions. Thus, these datasets are commonly used in the Person Re-ID research domain.

### 4.3. Comparison with State-of-the-Art Methods

We compared our EXAM with some state-of-the-art approaches. Our approach consistently outperforms the others on three datasets for either Rank 1 or mAP. The details are given as follows.

Market-1501: The comparison results are shown in Table 1. OSNet [38], a local-feature based method, achieves 94.8% and 84.9 % for Rank1 and mAP respectively. Our proposed method outperforms it by increasing 0.3% and 1.0% for Rank1 and mAP respectively. CAR [30], a state-of-the-arts global feedforward attention network has the best result for Rank1 result, while EXAM has a 1.2% improvement on mAP. In general, the proposed method achieved the outstanding performance.

DukeMTMC-reID: In Table 2, Rank1 accuracy and mAP on DukeMTMC-reID are reported. IANet [29] with a novel Interaction-and-Aggregation (IA) structure has the best performance of all other methods. In comparison, our method outperforms it by 0.3%.and 2.6% on Rank1 accuracy and mAP respectively. Our approach achieved the best results on this dataset.

CUHK03: This dataset uses the new protocol and employs two methods to annotate the bounding-boxes. As shown in Table 3, our method achieved Rank1=73.9%,mAP=68.6% on the labeled dataset and 69.2%,65.0% on detected dataset, which are better than all others for both types of annotation methods.

Figure 5 shows Top-10 ranking results for some query images on Market-1501. The results from first two queries demonstrate the model robustness: with just one back view query image, our method can find the correct identities with different postures. It is important to note that, some of the images are not even aligned correctly. Although the third query image is too vague to provide clear details, our approach can utilize horizontally partitioned part features, such as length of hair presented in the top parts, or the skin color of the legs in the bottom parts, to find matches and get satisfactory results. For the fourth query image, our framework is able to extract both global features: pedestrian’s black outfits, and local details: white backpack belt. Thus, all query image 4’s top 10 results contain those discriminative appearance elements.

### 4.4. Ablation Study

To further verify our framework, we conducted ablation studies on several variants with different combinations of embeddings and loss functions on the Market-1501 dataset. It should be noted that in each variant we only modified the relevant settings and kept the rest as the default.

**Figure 6:**
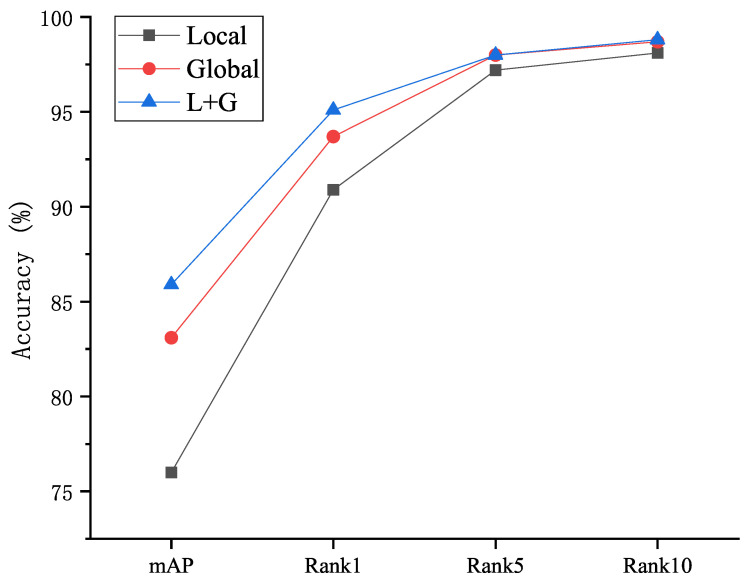
Performance of different branches on Market-1501 dataset.

First, we exclusively plugged the local or global embeddings into the model to test its performance individually. Figure 6 presents the results on mAP and accuracies of Rank 1, Rank 5 and Rank 10 respectively. We can see that, (1) using only local embeddings is not as effective as using only global embeddings. It means saliency and generality attentions derived from global features play more discriminative roles than the local features. (2) Given the high accuracy rate of only using the global embeddings, the recognition accuracy can be further improved by fusing both local and global embeddings. It validates the design of integration of global and local branches in our proposed EXAM framework.

**Figure 7:**
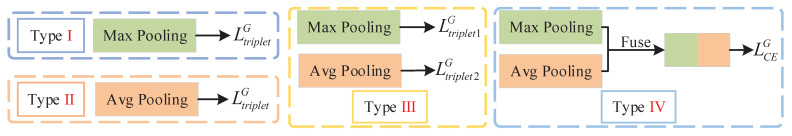
Some variants of the global branch.

Secondly, eight types of variants of the global branch with different combinations of embeddings and loss functions are shown in Table 4. Type 1 and Type 2 have the extreme and moderate embeddings respectively, where the triplet loss is applied for the training supervision. Type 3 merges both Type 1 and Type 2 and achieved higher accuracy on Rank 1 and mAP. Differing from Type 3, Type 4 fuses both extreme and moderate into a mixed embedding, and uses a single Softmax-based Cross-Entropy with Label Smoothing (defined in Equation (4)) as the loss function. Figure 7 shows the difference between Type 3 and Type 4. Both Rank 1 and mAP accuracies of Type 4 are 1+% better than Type 3. This set of variants indicates that, (1) using both extreme and moderate embeddings is better than using one alone; (2) using the fused embedding is more effective than using both separately. The best accuracy scores are achieved using the default global branch of EXAM where two separated embeddings and the fused embedding are all utilized. It implies both extreme and moderate embeddings bring positive attention cues for person Re-ID tasks.

Choosing the right loss functions for different embedding learning is crucial. CE loss is used to determine the feature representation to match the labeled target. Global variant Type 5 selects the triplet loss for the fused embedding. Without the supervision of CE loss, the learned feature representation of this variant lacks discriminative ability. Thus, its performance was deteriorated substantially compared with the default, i.e., decreased by 2.5%, 4.7% for Rank 1 and mAP respectively. Triplet loss provides an assistive role for feature representation learning, as it pushes the data from different identities apart in the feature space, while pulling the data closer if it belongs to the same person. Type 6 does not use any triplet loss, but instead uses CE loss for all three embeddings. Without the assistance from the triplet loss, the learning burden of the feature representation is increased. Thus, the performance of Type 6 is also decreased by 0.27% and 0.54% on Rank 1 and mAP respectively.

To further evaluate the effective usage of two types of loss functions, Type 8 switches positions of loss functions in the default EXAM, i.e., puts CE loss on both separated embeddings, and applies triplet loss on the fused embedding. This implies that it uses the fused embedding to learn the distance metric for data separation, and individual extreme and moderate embeddings to determine the feature representation learning. From the results, this variant has relatively poorer accuracy because it is difficult for the triplet loss to assist in data separation based on the mixed information. Meanwhile, separated extreme and moderate embeddings give limited information to CE loss for feature learning. Comparing Type 4 and Type 7, we also see that using more loss functions does not guarantee better performance, as Type 7 adds CE loss on the fused embedding, but received worse accuracy (down by −0.36%, −0.34% on Rank 1 and mAP).

**Figure 8:**
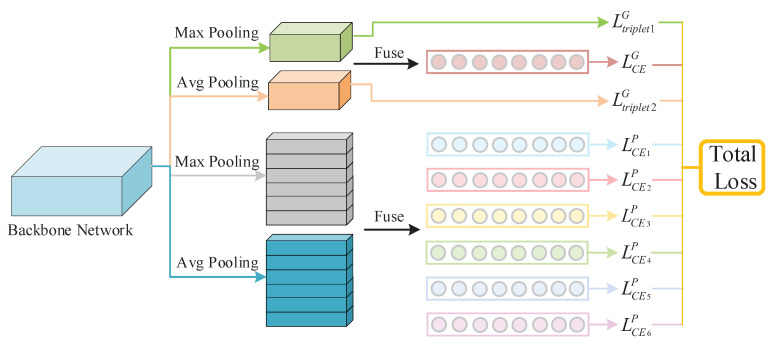
Variant of the local branch. The local extreme embeddings are brought into the framework.

**Figure 9:**
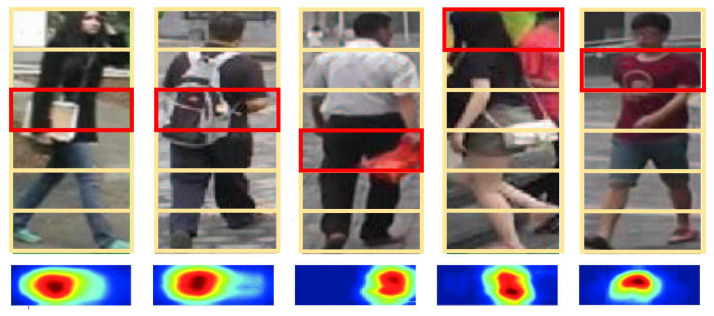
Saliency heat maps from local extreme embeddings. The second row lists the local salient heat maps derived from the red box of the bounding box in the first row.

Thirdly, similar to the global branch, additional local extreme embeddings are extracted and fused with the local moderate embeddings in the local branch. Figure 8 shows the structure of this variant. In the local branch, each partitioned part just contains partial information. Local extreme embedding only captures the saliency based on the incomplete features. For example, upper parts of a bounding box might be dominated by partial head or the background scene, while the middle or lower parts might contain unrelated occlusions. Figure 9 shows five examples, where the saliency heat maps are derived from corresponding local extreme embeddings. From the left to the right image, the local extreme (saliency) captures textbook, backpack, red plastic bag, background, and logo on the shirt respectively. None of those features are arguably important enough to describe the appearance. If those local extreme embeddings are brought into training framework, the feature learning process would be distracted, and often leads to wrong directions, resulting in worse identification accuracy. The Rank 1 accuracy of the structure in Figure 8 is down by 0.3% comparing with the proposed EXAM.

In summary, through the comparison of the above eight models, it is clear that the EXAM design is effective in person Re-ID.

## 5. Conclusions

In this paper, we propose an end-to-end EXAM framework learning Extreme and Moderate embeddings for Re-ID. The network has global and local branches. The global embeddings reflect the saliency and commonality of full human body appearance respectively. The local moderate embeddings capture the concepts of consistency and smoothness of body parts which adds robustness to the system to identify in cases of diverse posture variations. Both Extreme and Moderate embeddings from global and local views bring visual attention cues for discriminative feature learning under the deep supervision of multiple cross-entropy loss and triplet loss functions. The processes of attention deducing and discriminative feature learning are incorporated, and benefit from each other. From our comparative experiments and ablation studies, it is shown that EXAM is effective, and its learned feature representation reaches state-of-the-art performance. In future study, we plan to refine the weights of multi-loss to make it more effective.

## Figures and Tables

**Table 1:** Comparison results on Market-1501 dataset.

Method	Rank1	Rank2	Rank3	mAP
SVDNet [39]	82.3	92.3	95.2	62.1
MGCAM [11]	83.7	-	-	74.3
Triplet Loss [35]	84.9	94.2		69.1
AOS [40]	86.4	-	-	70.4
Dual [41]	91.4	-	-	76.6
Mancs [26]	93.1	-	-	82.3
CAMA [42]	94.7	-	-	84.5
MultiRegion [43]	66.4	85.0	90.2	41.2
PAR [44]	81.0	92.0	94.7	63.4
PDC [45]	84.4	92.7	94.9	63.4
AACN [46]	85.9	-	-	66.9
HA-CNN [25]	91.2	-	-	75.7
PCB [17]	92.3	97.2	98.2	77.4
PCB+RPP [17]	93.8	97.5	98.5	81.6
AANet [47]	93.9	-	98.5	83.4
Auto-ReID [48]	94.5	-	-	85.1
OSNet [38]	94.8	-	-	84.9
CAR [30]	96.1	-	-	84.7
**EXAM**	**95.1**	**98.0**	**98.8**	**85.9**

**Table 2:** Comparison results on DukeMTMC-reID dataset.

Method	Rank1	mAP
SVDNet [39]	76.7	56.8
AOS [40]	79.2	62.1
MLFN [49]	81.0	62.8
DuATM [50]	81.8	64.6
PCB+RPP [17]	83.3	69.2
PSE+ECN [21]	84.5	75.7
GP-reid [51]	85.2	72.8
CAMA [42]	85.8	72.9
CAR [30]	86.3	73.1
IANet [29]	87.1	73.4
**EXAM**	**87.4**	**76.0**

**Table 3:** Comparison results on the CUHK03 dataset. Besides the Rank1 accuracy and mAP presented in this table, our method has 87.0% and 92.6% accuracy for Rank5 and Rank10 on the labeled dataset, and 85.0% and 90.2% on the detected dataset.

Method	Labeled		Detected	
	Rank1	mAP	Rank1	mAP
PAN [52]	36.9	35.0	36.3	34.0
SVDNet [39]	40.9	37.8	41.5	37.3
DPFL [53]	43.0	40.5	40.7	37.0
HA-CNN [25]	44.4	41.0	41.7	38.6
MLFN [49]	54.7	49.2	52.8	47.8
DaRe+RE [54]	66.1	61.6	63.3	59.0
PCB+RPP [17]	-	-	63.7	57.5
Mancs [26]	69.0	63.9	65.5	60.5
DG-Net [55]	-	-	65.6	61.1
**EXAM**	**73.9**	**68.6**	**69.2**	**65.0**

**Table 4:** Variants of the global branch on the Market-1501 dataset.

Variant	Extreme		Moderate		Fusion		Accuracy(%)	
	Triplet	CE	Triplet	CE	Triplet	CE	Rank1	mAP
Type1	✓						93.4	82.8
Type2			✓				93.2	82.1
Type3	✓		✓				93.5	82.8
Type4						✓	94.6	84.4
Type5	✓		✓		✓		92.6	81.2
Type6		✓		✓		✓	94.9	85.1
Type7					✓	✓	94.1	84.8
Type8		✓		✓	✓		94.2	84.0
**EXAM**	✓		✓			✓	**95.1**	**85.9**

## Data Availability

Not applicable.

## References

[1] Li H., Kuang Z., Yu Z., Luo J. (2020). Structure alignment of attributes and visual features for cross-dataset person re-identification. Pattern Recognit..

[2] Zhao D., Wang H., Yin H., Yu Z., Li H. (2020). Person re-identification by integrating metric learning and support vector machine. Signal Process..

[3] Li H., Chen Y., Tao D., Yu Z., Qi G. (2021). Attribute-Aligned Domain-Invariant Feature Learning for Unsupervised Domain Adaptation Person Re-Identification. IEEE Trans. Inf. Forensics Secur..

[4] Li H., Xu J., Zhu J., Tao D., Yu Z. (2019). Top distance regularized projection and dictionary learning for person re-identification. Inf. Sci..

[5] Li H., Zhou W., Yu Z., Yang B., Jin H. (2020). Person re-identification with dictionary learning regularized by stretching regularization and label consistency constraint. Neurocomputing.

[6] Li H., Yan S., Yu Z., Tao D. (2019). Attribute-Identity Embedding and Self-supervised Learning for Scalable Person Re-Identification. IEEE Trans. Circ. Syst. Video Technol..

[7] Deng J., Dong W., Socher R., Li L.J., Li K., Fei-Fei L. Imagenet: A large-scale hierarchical image database. Proceedings of the IEEE Conference on Computer Vision and Pattern Recognition.

[8] Zhao Y., Lin J., Qi X., Xu X. (2019). HPILN: A feature learning framework for cross-modality person re-identification. IET Image Process..

[9] Zhang X., Luo H., Fan X., Xiang W., Sun Y., Xiao Q., Jiang W., Zhang C., Sun J. (2017). AlignedReID: Surpassing Human-Level Performance in Person Re-Identification. arXiv.

[10] Zhao H., Tian M., Sun S., Shao J., Yan J., Yi S., Wang X., Tang X. Spindle Net: Person Re-Identification with Human Body Region Guided Feature Decomposition and Fusion. Proceedings of the IEEE Conference on Computer Vision and Pattern Recognition.

[11] Song C., Huang Y., Ouyang W., Wang L. Mask-guided contrastive attention model for person re-identification. Proceedings of the IEEE Conference on Computer Vision and Pattern Recognition.

[12] Ning X.B., Yuan G., Yizhe Z., Christian P. Second-Order Non-Local Attention Networks for Person Re-Identification. Proceedings of the IEEE International Conference on Computer Vision.

[13] Shuang L., Slawomir B., Peter C., Xiaogang W. Diversity regularized spatiotemporal attention for video-based person re-identification. Proceedings of the IEEE Conference on Computer Vision and Pattern Recognition.

[14] Chen T., Ding S., Xie J., Yuan Y., Chen W., Yang Y., Ren Z., Wang Z. Abd-net: Attentive but diverse person re-identification. Proceedings of the IEEE International Conference on Computer Vision.

[15] Li W., Zhao R., Xiao T., Wang X. DeepReID: Deep Filter Pairing Neural Network for Person Re-Identification. Proceedings of the IEEE Conference on Computer Vision and Pattern Recognition.

[16] Yi D., Lei Z., Liao S., Li S.Z. Deep Metric Learning for Person Re-identification. Proceedings of the International Conference on Pattern Recognition.

[17] Sun Y., Zheng L., Yang Y., Tian Q., Wang S. Beyond Part Models: Person Retrieval with Refined Part Pooling (and A Strong Convolutional Baseline). Proceedings of the European Conference on Computer Vision.

[18] Tao D., Guo Y., Yu B., Pang J., Yu Z. (2018). Deep Multi-View Feature Learning for Person Re-Identification. IEEE Trans. Circ. Syst. Video Technol..

[19] Zheng Z., Zheng L., Yang Y. (2017). A Discriminatively Learned CNN Embedding for Person Reidentification. ACM Trans. Multimed. Comput. Commun. Appl..

[20] Li D., Chen X., Zhang Z., Huang K. Learning Deep Context-Aware Features Over Body and Latent Parts for Person Re-Identification. Proceedings of the IEEE Conference on Computer Vision and Pattern Recognition.

[21] Saquib Sarfraz M., Schumann A., Eberle A., Stiefelhagen R. A pose-sensitive embedding for person re-identification with expanded cross neighborhood re-ranking. Proceedings of the IEEE Conference on Computer Vision and Pattern Recognition.

[22] Wei L., Zhang S., Yao H., Gao W., Tian Q. (2019). GLAD: Global-Local-Alignment Descriptor for Scalable Person Re-Identification. IEEE Trans. Multimed..

[23] Yao H., Zhang S., Hong R., Zhang Y., Xu C., Tian Q. (2019). Deep Representation Learning with Part Loss for Person Re-Identification. IEEE Trans. Image Process..

[24] Jiang D., Qi G., Hu G., Mazur N., Zhu Z., Wang D. (2020). A residual neural network based method for the classification of tobacco cultivation regions using near-infrared spectroscopy sensors. Infrared Phys. Technol..

[25] Li W., Zhu X., Gong S. Harmonious Attention Network for Person Re-Identification. Proceedings of the IEEE Conference on Computer Vision and Pattern Recognition.

[26] Wang C., Zhang Q., Huang C., Liu W., Wang X. Mancs: A multi-task attentional network with curriculum sampling for person re-identification. Proceedings of the European Conference on Computer Vision.

[27] Chen G., Lin C., Ren L., Lu J., Zhou J. Self-Critical Attention Learning for Person Re-Identification. Proceedings of the IEEE International Conference on Computer Vision.

[28] Binghui C., Weihong D., Jiani H. Mixed high-order attention network for person re-identification. Proceedings of the IEEE International Conference on Computer Vision.

[29] Hou R., Ma B., Chang H., Gu X., Shan S., Chen X. Interaction-And-Aggregation Network for Person Re-Identification. Proceedings of the IEEE Conference on Computer Vision and Pattern Recognition.

[30] Zhou S., Wang F., Huang Z., Wang J. Discriminative feature learning with consistent attention regularization for person re-identification. Proceedings of the IEEE International Conference on Computer Vision.

[31] Hu G., Gao Q. A 3D gesture recognition framework based on hierarchical visual attention and perceptual organization models. Proceedings of the IEEE 21st International Conference on Pattern Recognition (ICPR2012).

[32] Bai X., Yang M., Huang T., Dou Z., Yu R., Xu Y. (2020). Deep-Person: Learning discriminative deep features for person Re-Identification. Pattern Recogn..

[33] Hu G., Dixit C., Luong D., Gao Q., Cheng L. Salience Guided Pooling in Deep Convolutional Networks. Proceedings of the IEEE International Conference on Image Processing (ICIP).

[34] Szegedy C., Vanhoucke V., Ioffe S., Shlens J., Wojna Z. Rethinking the Inception Architecture for Computer Vision. Proceedings of the IEEE Conference on Computer Vision and Pattern Recognition.

[35] Hermans A., Beyer L., Leibe B. (2017). In Defense of the Triplet Loss for Person Re-Identification. arXiv.

[36] Zhong Z., Zheng L., Kang G., Li S., Yang Y. (2017). Random Erasing Data Augmentation. arXiv.

[37] Felzenszwalb P., McAllester D., Ramanan D. A discriminatively trained, multiscale, deformable part model. Proceedings of the IEEE Conference on Computer Vision and Pattern Recognition.

[38] Zhou K., Yang Y., Cavallaro A., Xiang T. Omni-Scale Feature Learning for Person Re-Identification. Proceedings of the IEEE International Conference on Computer Vision.

[39] Sun Y., Zheng L., Deng W., Wang S. Svdnet for pedestrian retrieval. Proceedings of the IEEE International Conference on Computer Vision.

[40] Huang H., Li D., Zhang Z., Chen X., Huang K. Adversarially occluded samples for person re-identification. Proceedings of the IEEE Conference on Computer Vision and Pattern Recognition.

[41] Du Y., Yuan C., Li B., Zhao L., Li Y., Hu W. Interaction-aware spatio-temporal pyramid attention networks for action classification. Proceedings of the European Conference on Computer Vision.

[42] Yang W., Huang H., Zhang Z., Chen X., Huang K., Zhang S. Towards Rich Feature Discovery with Class Activation Maps Augmentation for Person Re-Identification. Proceedings of the IEEE Conference on Computer Vision and Pattern Recognition.

[43] Ustinova E., Ganin Y., Lempitsky V. Multi-region bilinear convolutional neural networks for person re-identification. Proceedings of the IEEE International Conference on Advanced Video and Signal Based Surveillance.

[44] Zhao L., Li X., Zhuang Y., Wang J. Deeply-Learned Part-Aligned Representations for Person Re-Identification. Proceedings of the IEEE International Conference on Computer Vision.

[45] Su C., Li J., Zhang S., Xing J., Gao W., Tian Q. Pose-Driven Deep Convolutional Model for Person Re-Identification. Proceedings of the IEEE International Conference on Computer Vision.

[46] Xu J., Zhao R., Zhu F., Wang H., Ouyang W. Attention-Aware Compositional Network for Person Re-Identification. Proceedings of the IEEE Conference on Computer Vision and Pattern Recognition.

[47] Tay C.P., Roy S., Yap K.H. AANet: Attribute Attention Network for Person Re-Identifications. Proceedings of the IEEE Conference on Computer Vision and Pattern Recognition.

[48] Quan R., Dong X., Wu Y., Zhu L., Yang Y. Auto-ReID: Searching for a Part-Aware ConvNet for Person Re-Identification. Proceedings of the IEEE International Conference on Computer Vision.

[49] Chang X., Hospedales T.M., Xiang T. Multi-Level Factorisation Net for Person Re-Identification. Proceedings of the IEEE Conference on Computer Vision and Pattern Recognition.

[50] Si J., Zhang H., Li C.G., Kuen J., Kong X., Kot A.C., Wang G. Dual attention matching network for context-aware feature sequence based person re-identification. Proceedings of the IEEE Conference on Computer Vision and Pattern Recognition.

[51] Xiong F., Xiao Y., Cao Z., Gong K., Fang Z., Zhou J.T. (2018). Towards good practices on building effective cnn baseline model for person re-identification. arXiv.

[52] Zheng Z., Zheng L., Yang Y. (2019). Pedestrian Alignment Network for Large-scale Person Re-Identification. IEEE Trans. Circ. Syst. Video Technol..

[53] Chen Y., Zhu X., Gong S. Person re-identification by deep learning multi-scale representations. Proceedings of the IEEE International Conference on Computer Vision.

[54] Wang Y., Wang L., You Y., Zou X., Chen V., Li S., Huang G., Hariharan B., Weinberger K.Q. Resource aware person re-identification across multiple resolutions. Proceedings of the IEEE Conference on Computer Vision and Pattern Recognition.

[55] Zheng Z., Yang X., Yu Z., Zheng L., Yang Y., Kautz J. Joint discriminative and generative learning for person re-identification. Proceedings of the IEEE Conference on Computer Vision and Pattern Recognition.

